# The Prevalence of Temporomandibular Disorders and Dental Attrition Levels in Patients with Posterior Crossbite and/or Deep Bite: A Preliminary Prospective Study

**DOI:** 10.1155/2021/8827895

**Published:** 2021-02-12

**Authors:** Naser Khayat, Efraim Winocur, Ron Kedem, Orit Winocur Arias, Ayman Zaghal, Nir Shpack

**Affiliations:** ^1^Maurice and Gabriela Goldschleger School of Dental Medicine, Tel Aviv University, Tel Aviv-Yafo, Israel; ^2^Department of Oral Rehabilitation, Maurice and Gabriela School of Dental Medicine, Tel Aviv University, Tel Aviv-Yafo, Israel; ^3^Academic Branch of the IDF Medical Corps, Tel Hashomer, Israel; ^4^Department of Oral Pathology and Oral Medicine, Maurice and Gabriela Goldschleger School of Dental Medicine, Tel Aviv University, Tel Aviv-Yafo, Israel; ^5^Department of Oral and Maxillofacial Surgery, Al Quds University, Jerusalem, Israel; ^6^Department of Orthodontics, Maurice and Gabriela Goldschleger School of Dental Medicine, Tel Aviv University, Tel Aviv-Yafo, Israel

## Abstract

**Background:**

The prevalence of various temporomandibular disorders (TMD) and the severity of attrition in patients with either bilateral or unilateral deep bite and/or posterior crossbite has not been established, nor has the effect of one year of orthodontic treatment on TMD.

**Methods:**

Of 310 patients presenting with suspected TMD, 160 were diagnosed with various TMD and 150 were TMD-free. Diagnosis was according to the Axis I of the Diagnostic Criteria for TMD. All participants underwent a dental examination, and 100 patients were reevaluated after one year of orthodontic treatment. Fisher's exact test and the proportion test with Bonferroni's correction were used for the categorical univariate analysis.

**Results:**

There was a significant association (*P* < 0.001) between deep bite and dental attrition (wear), but not between crossbite and/or deep bite in patients diagnosed with either painful TMD or disc displacement. The risk of sustaining painful TMD when crossbite presented simultaneously on the anterior and the posterior dentition was 2.625-fold greater than when it presented with a normal bite, although this difference was not significant (*P*=0.286) due to the lack of statistical power. There was no significant sex-related association between the occurrence of either painful TMD or disc displacement. A reduction in TMD findings was demonstrated after one year of treatment, but no statistical power was reached due to the small sample size.

**Conclusions:**

Deep bite may be related to dental wear but not to pain from TMD and/or disc displacement. Only crossbite that presents simultaneously on the anterior and the posterior dentition (mixed X-bite) may have some effect on the level of pain in TMD, but not on in the prevalence of disc displacement. Confirmation of these conclusions by well-designed studies on larger patient groups is warranted. There was a clinically significant improvement in TMD findings after one year of treatment.

## 1. Introduction

The term “temporomandibular disorders” (TMDs) refers to a set of clinical problems that involve the masticatory musculature, the temporomandibular joint (TMJ), and associated structures or both [1]. It has been identified as the leading cause of nondental pain in the orofacial region and is considered a subclass of musculoskeletal disorders [2]. Several investigations on the relationship between occlusal factors and TMDs have been carried out. List et al.'s [3] case control study found that psychosocial factors, somatic complaints, and emotional problems seem to play a more prominent role than dental factors in adolescents with TMDs. Although occlusion has been hypothesized as an etiological or perpetuating cofactor, the degree to which it plays a role has not been definitively delineated [4]. Pullinger and Seligman [5] identified some occlusal features as being potentially related to TMDs, including unilateral lingual crossbite and deep bite. In Magnusson et al.'s study which extended over a 20-year period, a unilateral posterior crossbite was considered as being a possible local risk factor for the development of TMDs [4]. Others [6], however, rejected this latter hypothesis, showing that TMDs are either weakly or not at all associated with posterior crossbite.

Extreme deep bite values have shown an association with TMDs, which is a necessary but not a sufficient sole criterion for a causal relationship [7]. In addition, some researchers are of the opinion that occlusion may be an important factor in causing TMDs [8], while most reviews concluded that malocclusion does not cause TMDs [9].

Tooth wear is a multifactorial condition leading to the progressive loss of dental hard tissue through three processes: attrition (loss of substance on opposing occlusal units caused by tooth-to-tooth contact), abrasion (wear produced by interaction between teeth and artificial materials), and erosion (dissolution of teeth by acidic substances) [10]. Attrition is the most frequently mentioned sign of bruxism [11], although there is considerable controversy about the correlation between bruxism and tooth wear. Some investigators suggest that bruxism causes attrition [12], but others have not found the tooth wear status to be predictive of ongoing bruxism levels [13]. Lavigne et al. are of the opinion that tooth wear should not be considered a specific marker of bruxism since the cause (bruxism) and the effect (tooth wear) could have occurred long before the dental wear had been diagnosed [14].

Given all of these dissenting opinions and claims taken together, the aims of this study were twofold: to assess the occurrence of different TMDs and the presence and severity of attrition in patients with posterior crossbite and/or deep bite and to assess the effect of 1-year orthodontic treatment on TMDs. The null hypotheses of the study were as follows. (1) The prevalence of posterior crossbite and/or deep bite will be significantly greater among patients with TMDs. (2) The prevalence and severity of dental attrition will be significantly greater among patients with posterior crossbite and/or deep bite. (3) The incidence of TMDs will remain unchanged after one year of orthodontic treatment.

## 2. Materials and Methods

### 2.1. Study Population

The study population was composed of two cohorts that included a total of 310 patients with an age range of 11–49 years. There were 153 males (49.4%) with a mean age of 16.8 years (95% confidence interval (CI): 16.1, 17.5) and 157 females (50.6%) with a mean age of 19.4 years (95% CI: 18.2, 20.6). The sample was taken from two sources in 2018: patients who were referred to the Postgraduate Orthodontic Clinic, Department of Orthodontics, The Maurice and Gabriela Goldschleger School of Dental Medicine, Tel Aviv University, Tel Aviv, Israel (71 patients), and those referred to a private orthodontic clinic located in East Jerusalem (89 patients). All of the participants (Table 1) were diagnosed according to the DC/TMD (AXIS I) (http://rdc-tmdinternational.org) [15].

The TMD group consisted of 160 patients. It was further divided into (1) the painful TMD group (TMD pain: 53 patients; 17%) defined by the presence of at least one of the following: myalgia, myofascial pain, headache attributed to TMD, or arthralgia; (2) the disc displacement (DD) group (54 patients; 18%): defined by the presence of at least one of the following: DD with reduction, DD with reduction with intermitting locking, and DD without reduction; (3) the mixed group (mixed TMD: 53 patients; 17%) defined as patients that present with at least one diagnosis belonging to the TMD pain group, and at least one diagnosis belonging to the DD group. The TMD-free group (FREE TMD) consisted of 150 orthodontic patients that were found not to have any TMDs.

### 2.2. Initial Clinical Examination

#### 2.2.1. Temporomandibular Clinical Examination and Diagnosis

All patients were examined by the examining investigator (NK), who had been previously calibrated according to the training practice session of the International Consortium for TMD (http://rdc-tmdinternational.org) [15].

#### 2.2.2. Dental Clinical Examination and Diagnosis

The examining investigator (NK), an orthodontic specialist, examined all patients. A posterior crossbite (X-bite) was defined when one or more of the teeth of the posterior dental group (from canine to second molar) were found to present at least one cusp wide buccolingual or buccopalatal relationship, with one or more opposing teeth in intercuspal position [16]. An anterior crossbite was defined when one or more maxillary anterior teeth are palatally positioned relative to the mandibular anterior teeth [17].

The patients' final diagnosis was no X-bite (NoXB), anterior X-bite (AntXB) only, posterior X-bite (PostXB), which included an X-bite unilaterally or bilaterally, and mixed X-bite (MIXXB), which included AntXB and PostXB.

An overbite was defined by the vertical overlap of lower incisors by the upper incisors. An overbite was considered normal when 1/3 of the lower incisor was covered by the upper incisor (grade 0). A vertical overlap ranging from 1/3 to less than 2/3 was considered a mild deep bite (grade 1), a vertical overlap from 2/3 to less than full length of the lower incisor was considered a moderate deep bite (grade 2), and a vertical overlap equal to or more than the full length of a lower incisor was considered a severe deep bite (grade 3) [18]. A negative overbite was considered an open bite. Patients were finally categorized as having a normal-to-mild = grade 0–1 = NorMil or a moderate-to-severe = grade 2–3 = ModSev.

Tooth attrition was assessed on a tooth-by-tooth basis according to Lobbezo and Naeije [19] and rated according to a 5-point ordinal scale (0–4), where grade 0 = no wear, grade 1 = visible wear within the enamel only, grade 2 = visible wear with dentin exposure and mild-to-moderate loss of clinical crown height (≤1/3), grade 3 = significant loss of crown height >1/3 but <2/3, and grade 4 = loss of crown height (≥2/3). All of the teeth, including the anterior ones, were included in the analysis. Wear was calculated for each subject as an average score [20]. Teeth affected by erosion were excluded. The final analysis was performed according to the European Consensus Statement (2017) [21] as low wear = grades 0–2, and high wear = grades 3-4.

### 2.3. Repeat Clinical Examination

The first 1/3 of the study patients to be enrolled were reevaluated during a routine orthodontic appointment one year from the initial examination. Each was fully examined and a diagnosis was again arrived at according to the DC/TMD and by the same clinician. The examiner was blind to the initial diagnosis in order to prevent bias.

### 2.4. Ethical Concerns

Approval to conduct this study was obtained from the Tel Aviv University Institutional Ethical Committee (no.: 20170529 12272191) on May 29, 2017. Written and oral informed consent was provided by all of the participants or the legal guardian. The study was self-funded by the authors.

### 2.5. Statistical Methods

A univariate analysis followed by Fishers' exact test was used for the descriptive data. A proportion test with Bonferroni correction was applied as appropriate for categorical variables. ANOVA with Tukey's post hoc test was employed for comparing the patient's age and sex of the X-bite and the overbite groups (without the Tukey's post hoc test). The variously diagnosed groups were analyzed for each category variable: “TMD pain,” “DD,” and “mixed TMD were compared to the reference group “FREE TMD” by using Fisher's exact test and the proportion test with Bonferroni's correction. The significance level of the result was *α* = 0.05. The data were analyzed using IBM SPSS statistics version 23.0. (SPSS, Inc., Chicago, IL USA).

## 3. Results

### 3.1. Sampling

**Figure 1:**
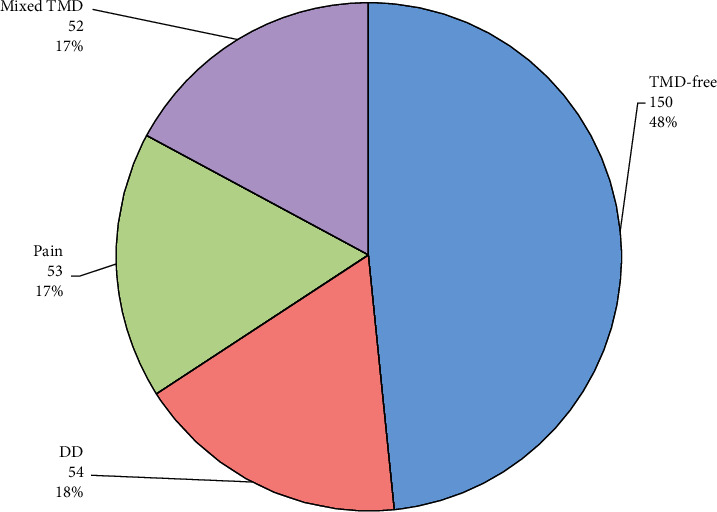
Distribution of the temporomandibular disorders (TMD) groups. DD = disc displacement; pain = painful TMD; mixed TMD = DD + Pain.

Around one-half (49.4%) of the 310 patients included in this study were men. The FREE TMD group comprised about one-half (48%) of the study participants. In the TMD group, 18% were diagnosed with DD alone (the DD group), 17% had temporomandibular pain alone (the TMD pain group), and 17% of the patients had both DD and temporomandibular pain (the mixed TMD group) (Figure 1).

**Figure 2:**
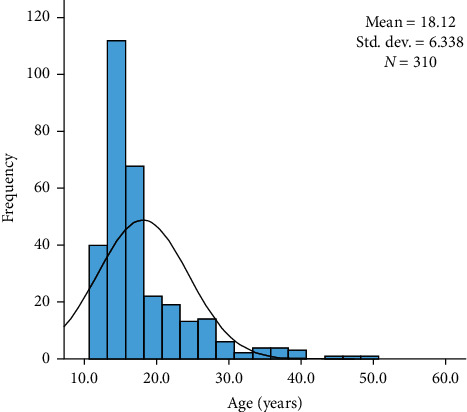
Age distribution of the participants.

The average age of the study participants was 18.1 years (range 11–49 years; Figure 2), but since the age demonstrates a right skewed distribution (*P* < 0.001 in Kolmogorov–Smirnov with Lilliefors Significance Correction), which invalidates the use of a parametric test, the median 16.0 years denotes better the center of the sample group. To allow analysis on the age variable, it was coded into two age groups with a cut-point using ROC analysis with dental wear.

**Figure 3:**
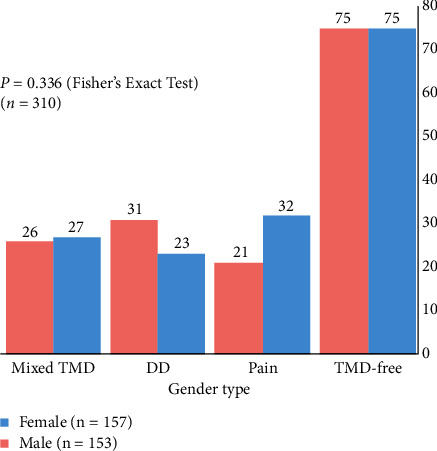
Distribution of TMD groups by sex. DD = disc displacement; TMD = temporomandibular disorders.

The sampling of sex type did not differ among the TMD groups (*P*=0.336; Figure 3), but the young age group comprised significantly more males (57.1%; *P* < 0.001) than the elder group (17.5–50.0), as expected in an orthodontic clinic (Table 1).

The prevalence of TMD pain in the patients with mixed XB was 21%, while it was only 16% in those with NoXB (a relative risk of 1.3125) (Table 2). The prevalence of DD was 11% in patients that exhibited mixed XB, while it was 22% in those with NoXB (a relative risk of 0.50). This meant that the risk of belonging to the TMD pain group compared to the DD group in the mixed XB group was 2.625-fold greater than for the NoXB. Nevertheless, due to the lack of power, this difference was not found to be significant (*P*=0.286) (only three patients had both mixed XB and DD). The prevalence of mixed TMD in patients with mixed XB was 21% compared to 13% among those with NoXB (a relative risk of 1.6154). The risk of belonging to the mixed TMD group compared to the DD group in those with mixed XB was 3.231-fold greater than in patients with NoXB, but again the difference was not found to be significant (*P*=0.146).

There was a significant (*P* < 0.001) dependency between deep bite and wear (Table 3): 46 (17%) of the 275 participants were found to have had a ModSev bite. Of them, 38% exhibited extensive wear, and only 13% patients had low levels of wear. The odds ratio according to the Mantel–Haenszel common estimate analysis was 4.21 (95% CI: 2.05, 8.62).

### 3.2. Age, TMD, and Dental Wear

**Figure 4:**
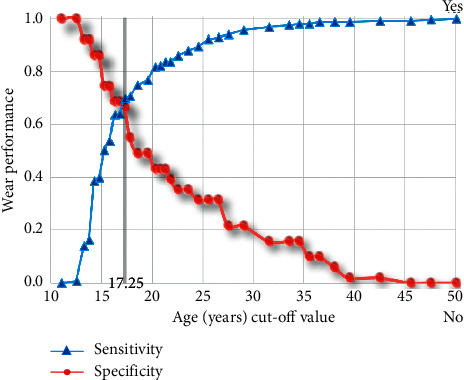
ROC analysis for age cutpoint via dental wear.

Due to the sample size, the lack of power was prevented from this study to assess the influence of age on patients with crossbite or deep bite in the occurrence of TMD, and it was not the aim of the study. It was found that 57.1% of the male are in the range of 11.0–16.9 age group which is significantly higher than the 17.5–50.0 age group (35.7%). The age cut-point when wear sensitivity and specificity is minimal is 17.5 years (the area under the curve is 0.699, 95% CI [0.617–0.780]; asymptotic significance when null hypothesis, true area = 0.5, is <0.001) (Figure 4). When computing dichotomously the two-age group (<17.5; and ≥17.5), and cross-check it with each TMD group, it was found that age is not an influence factor of any of the TMD groups (Table 4), but it is a significant factor in the developing of dental wear (*P* < 0.001, Table 5). In other words, it can be concluded that age is not an influence factor to the research variables.

### 3.3. TMD Diagnoses at Reevaluation

The interval between the initial diagnosis and the reevaluation was 12.21 ± 0.51 months (range of 12–14). Comparison of the results of the initial diagnosis with the results of the reevaluation revealed a clear-cut improvement after one year of orthodontic treatment. By the time of the follow-up evaluation at one year, the TMD-free group increased from 32% to 73%, while the distribution of the TMD diagnoses decreased as follows: the DD group decreased from 21% to 16%, the TMD pain group decreased from 28% to 7%, while the mixed TMD group decreased from 19% to 4%. The McNemar–Bowker Test of Symmetry was used for testing correlated proportions of the initial diagnosis and of the reevaluation (*P* < 0.0001). In addition, proportion tests with Bonferroni correction were calculated for each cell and the results are displayed in Table 6. Achieving statistical power for assessing the results required a sample size of 300.

## 4. Discussion

We had hypothesized that the prevalence of posterior crossbite and/or deep bite will be greater among patients with TMDs, that the prevalence and severity of dental attrition will be significantly greater among patients with posterior crossbite and/or deep bite, and that the incidence of TMDs will remain unchanged after 1 year of orthodontic treatment. These null hypotheses were only partially rejected by the findings of this study.

There was no significant sex-related association (*P*=0.336) between the occurrence of TMDs. These findings are in contrast to those of Rushdi Khayat et al. [22] who studied the prevalence of TMDs, occlusal diagnosis, and bruxism in the general population and found that there was a significant sex difference (more among females) in the prevalence of painful TMDs. They also oppose those of Bagis et al. [23] who found that females had TMD signs and symptoms more frequently than males in their study population. The present findings are, however, in accordance with Khayat et al. [24] who reported that there is no significant association between the sex of the patient and the occurrence of either pain TMDs or DD. It was also found that age has no influence to the research TMD variables, but it is a significant factor in the developing of dental wear. This finding has to be cautiously considered due to the lack of power in the present sample.

Thilander et al. [25] found that TMDs were significantly associated with posterior crossbite, and those authors recommended that such malocclusions should be treated orthodontically at an early age to eliminate the traits of the anomaly. Additionally, Sonnesen et al. [26] observed that signs and symptoms of TMDs were significantly associated with malocclusions, such as unilateral posterior crossbite. Alarcon et al. [27] suggested that the altered morphological/occlusal relationship between the upper and lower dentition may result in right-to-left-side differences in the masticatory muscles and the condyle–fossa relationship and that the asymmetric activity of the masticatory muscles could therefore be the source of their tenderness. In contrast, Al-Ani [28] and Špalj et al. [29] reported that signs and symptoms of TMDs seemed to be poorly related to malocclusions. The present study found that a painful TMD diagnosis was prevalent only among those patients who exhibited both anterior and posterior crossbite (the mixed XB group) compared to patients with a normal bite (the NoXBite group). As far as we know, this is the first time this population of patients had been analyzed since the reports in the literature deal with cases of either anterior or posterior crossbite but not with both appearing concomitantly. In other words, in the current work, only severe cases of full mouth crossbite (anterior and posterior) may be a risk factor for the development of temporomandibular pain. This difference was not found to be significant (*P*=0.286) due to lack of power (only three of the study patients had both mixed XB and DD), so these figures should be interpreted with caution until well-designed studies with larger samples confirm them.

The present study demonstrated a significant dependency between deep bite and dental wear. These findings are in accordance with Richard et al. [30] who found that the attrition score tends to increase with the bite depth. They also agree with Grzegocka et al. [31], who concluded that deep bite in association with “tight incisal occlusion” represents an additional risk factor for dental wear, advising that orthodontic treatment is aimed at modification of the occlusion. These findings, however, are in opposition to the results of Seligman et al. [12] who reported that dental attrition in a nonpatient population was not associated with either signs or symptoms of TMDs or with occlusal factors.

We observed a remarkable reduction in TMDs when comparing the initial TMD diagnoses with the results of the reevaluation after one year of orthodontic treatment. Unfortunately, sample size precluded achieving statistical power and a study on a larger sample study is warranted to address this issue. These findings are in accordance with Tecco et al. [32] who observed a significant improvement in myofascial pain syndrome (muscular pain) after treating the malocclusion by means of a fixed orthodontic appliance. Those authors hypothesized that orthodontic therapy could allow the improvement of the maxillomandibular relationship, thus improving the function of the related muscles. Also, Henrikson et al. [33] found that the prevalence of pain upon mandibular movement and of tenderness to palpation of the masticatory muscles was significantly less common during and after orthodontic treatment than before. In contrast, Nielsen et al. [34] evaluated the effect of orthodontic treatment on the functional status of the masticatory system and found that tenderness on palpation of the musculature and the TMJ capsule were generally more prevalent among orthodontically treated subjects. The improvement that we observed in the 1-year reevaluation can be due to the fact that the teeth are sensitive during the active phase of the orthodontic treatment, especially during loading [35]. That would explain why patients report some reduction of bruxism during the period of orthodontic treatment [36]. This reduction in the muscular hyperactivity and in the bruxist's grinding and clenching of the teeth might have a favorable effect on painful TMD and even on the occurrence of clicks, as discussed below.

The reduction in the occurrence of DD may be due the fact that the sensitivity of a DD diagnosis without magnetic resonance imaging is only 0.34, which raises some doubt about the accuracy of the diagnosis. It must be kept in mind that clicks may be caused by factors other than disc position [37]. Moreover, awake clenching can cause temporary disc adhesion [38] which is released when moving the mandible (opening or contralateral movement). The energy invested is expressed as a click [39]. In other words, the reduction in the DD group at reevaluation may express a reduction in awake bruxism. Further study that includes orthodontic patients definitively diagnosed as having awake bruxism is warranted to clarify this possibility.

## 5. Conclusions

1. Deep bite and age may be related to dental wear but not to pain associated with TMDs and/or DD.
2. Only crossbite presenting concomitantly on both the anterior and the posterior dentition (the Mixed X-bite group) may have some effect on the pain associated with TMDs, but not on the prevalence of DD. Confirmation of these conclusions by well-designed studies on larger patient groups is warranted.
3. A clinically significant improvement of TMD findings was found after one year of orthodontic treatment.

## Figures and Tables

**Table 1:** Age group by sex type.

Testing group	Age group (years)	Male	Female	Fisher's exact test exact sig. (2-sided)	Odds ratio
Sex	11.0–16.9	57.1%	42.9%	*P* < 0.001	1.598 95% CI [1.212–2.106]
		198	85		
	17.5–50.0	35.7%	64.3%		
		112	72		

**Table 2:** Dental diagnoses at initial evaluation (according to TMD groups).

		Initial diagnosis groups				Total	*P*
		TMD-free (control)	Pain	DD	Mixed TMD		
	Total	150 (48%)	53 (17%)	54 (17%)	53 (17%)	310 (100%)	
X-bite, *n*	No X-bite	88_a, b_ (49%)	29_a, b_ (16%)	39_b_ (22%)	23_a_ (13%)	179 (100%)	0.294
	Anterior X-bite	9 (43%)	3 (14%)	4 (19%)	5 (24%)	21 (100%)	
	Posterior X-bite	40 (49%)	15 (18%)	8 (10%)	19 (23%)	82 (100%)	
	Mixed X-bite	13 (46%)	6 (21%)	3 (11%)	6 (21%)	28 (100%)	

Open bite, *n*	No open bite	117 (48%)	42 (17%)	41 (17%)	42 (17%)	242 (100%)	0.713
	Anterior open bite	20 (49%)	6 (15%)	8 (20%)	7 (17%)	41 (100%)	
	Posterior open bite	6 (46%)	3 (23%)	4 (31%)	0 (0%)	13 (100%)	
	Mixed open B	7 (50%)	2 (14%)	1 (7%)	4 (29%)	14 (100%)	

Overbite, *n*	Normal or mild	110 (48%)	42 (18%)	42 (18%)	35 (15%)	229 (100%)	0.547
	Deep bite	24 (52%)	6 (13%)	8 (17%)	8 (17%)	46 (100%)	

Wear, *n* (erosion excluded)	Low	127 (49%)	45 (17%)	46 (18%)	41 (16%)	259 (100%)	0.856
	High	23 (45%)	8 (16%)	8 (16%)	12 (24%)	51 (100%)	

TMD = temporomandibular disorders; pain = pain = painful TMD; DD = disc displacement; mixed TMD = DD + PAIN. _a, b_Each subscript letter denotes a subset at reevaluation categories whose column proportions do not differ significantly from each other at the 0.05 level with Bonferroni correction. So, _a_ is a significantly different proportion comparing to _b_ proportion with Bonferroni correction. _a, b_Proportions are overlapped. The proportion of normal bite is compared to not-normal bites.

**Table 3:** Low wear compared to high wear (erosion excluded) versus deep bite.

		Deep bite			*P*
		Normal or mild	Moderate or severe	Total	
Total, *n*		229 (83%)	46 (17%)	275 (100%)	
Dental wear^*∗*^	Low	201 (**87**%)	29 (13%)	230 (100%)	<0.001
	High	28 (62%)	17 (**38**%)	45 (100%)	

^*∗*^35 patients with eroded dentition were excluded. Bold indicates no conflicted result.

**Table 4:** Age group by TMD groups and male sex type.

Testing group	Age group (years)	Yes (%)	*N*	Fisher's exact test exact sig. (2-sided)	Odds ratio comparing to TMD-free
Pain	11.0–16.9	26.4	125	0.520	1.0296 95% CI [0.638–1.661]
	17.5–50.0	26.4	78		

DD	11.0–16.9	29.8	131	0.102	1.449 95% CI [0.860–2.442]
	17.5–50.0	20.5	73		

Mixed TMD	11.0–16.9	27.0	126	0.424	1.094 95% CI [0.674–1.775]
	17.5–50.0	24.7	77		

TMD = temporomandibular disorders; pain = pain = painful TMD; DD = disc displacement; mixed TMD = DD + pain.

**Table 5:** Age group by dental wear.

Testing group	Age group (years)	Severe	Not severe (erosion excluded)	Fisher's exact test exact sig. (2-sided)	Odds ratio
Dental wear	11.0–16.9	8.6%	91.4%	*P* < 0.001	4.641 95% CI [2.448–8.800]
		17	181		
	17.5–50.0	30.4%	69.6%		
		51	78		

**Table 6:** Comparison of initial diagnoses with reevaluation.

			Diagnoses at reevaluation				Initial diagnosis
			TMD-free	TMD pain	DD	Mixed group	
Initial diagnoses	TMD-free	Count	25_a, b_	0_b_	4_a, b_	3_a_	32
		% within initial diagnosis	78.1%	0.0%	12.5%	9.4%	100.0%
	TMD pain	Count	22_a, b_	5_b_	1_a_	0_a, b_	28
		% within initial diagnosis	78.6%	17.9%	3.6%	0.0%	100.0%
	DD^*∗∗*^	Count	15_a_	0_a_	6_a_	0_a_	21
		% within initial diagnosis	71.4%	0.0%	28.6%	0.0%	100.0%
	Mixed group	Count	11_a_	2_a_	5_a_	1_a_	19
		% within initial diagnosis	57.9%	10.5%	26.3%	5.3%	100.0%

Total		Count	73	7	16	4	100
		% within initial diagnosis	73.0%	7.0%	16.0%	4.0%	100.0%

TMD = temporomandibular disorders; pain = pain = painful TMD; DD = disc displacement; mixed TMD = DD + pain. _a, b_Each subscript letter denotes a subset at reevaluation categories whose column proportions do not differ significantly from each other at the 0.05 level with Bonferroni correction. So, _a_ is a significantly different proportion comparing to _b_ proportion with Bonferroni correction. _a, b_Proportions are overlapped.

## Data Availability

The data are available upon reasonable request from the corresponding author.
